# Ageing Well in Small Villages: What Keeps Older Adults Happy? Environmental Indicators of Residential Satisfaction in Four Dutch Villages

**DOI:** 10.3390/ijerph19073922

**Published:** 2022-03-25

**Authors:** Nienke J. A. Moor, Kim Hamers, Masi Mohammadi

**Affiliations:** 1 Research Group Architecture in Health, HAN University of Applied Sciences, 6846 CC Arnhem, The Netherlands; Kim.Hamers@han.nl (K.H.); or m.mohammadi@tue.nl (M.M.); 2 Smart Architectural Technologies, Eindhoven University of Technology, 5644 MB Eindhoven, The Netherlands

**Keywords:** residential satisfaction, liveability, older adults, small villages, living environment, mixed methods

## Abstract

This article aims to contribute to the existing literature about liveability in rural areas by explicitly focusing on the level of residential satisfaction of older adults (55+) in four small Dutch villages. We strive not only to identify the key indicators of residential satisfaction among older villagers but also to better understand how these indicators affect their (daily) life. Moreover, in line with the person–environment fit tradition, we differentiate according to the capabilities and vulnerabilities of older villagers. To this end, we use a mixed-method approach, in which we combine survey data with qualitative data collected with photovoice in the four villages. The findings indicate that older adults’ perceptions of spatial, social and functional aspects of the living environment are related to the degree of residential satisfaction overall. However, these perceptions appear to be strongly intertwined, especially perceptions about spatial characteristics, local identity and connectedness. Older adults who are hindered by health problems in undertaking daily activities experience a lower level of person–environment fit, which is reflected in a lower level of residential satisfaction. However, this relationship between subjective health and residential satisfaction can only be partially explained by different perceptions of the spatial, social and functional environment.

## 1. Introduction

As recent demographic developments in rural areas such as ageing and population decline have created a challenging context for ageing in place, there is a growing concern for the housing situation of older adults in the countryside. Although the proportion of Europeans living in rural areas is expected to decrease with time, the percentage of older adults in rural areas compared to urban areas is expected to increase [1]. A UNECE policy brief on ageing (No. 18, March 2017) pointed out that many UNECE countries experience pronounced population ageing, especially in rural regions. As a consequence, older adults in these regions with age-related disabilities may be hindered by a relatively low number of potential informal caregivers, reduced mobility and a limited range of facilities, including health and care services. Therefore, strategies need to be designed in order to narrow the gap between this urban and rural divide and to optimize the (social) strengths of the countryside to promote healthy ageing. 

Socio-spatial characteristics of the living environment can have a large impact on the well-being, residential satisfaction and health status of older adults [2,3,4,5,6,7] and can differ between urban and rural areas. Although the urban–rural distinction in ageing research is incorporated within environmental gerontology, there is an ongoing debate about its added value and quality [8,9]. An important point of criticism is that the classic distinction between urban and rural, which has been used several times in gerontological research, is too simplistic, as it neglects diversity within urban and rural settings [10]. From this perspective, ageing research should provide insight into the types of (rural) communities in which older adults are most satisfied.

### 1.1. Small (Remote) Villages in The Netherlands

Moreover, in the Netherlands, there are concerns about older adults living in small (remote) villages, even though rural and urban areas are strongly interconnected, and geographical distances are relatively small. It is assumed that the socio-spatial characteristics of the countryside can affect the liveability of older residents. A series of studies that monitored the level of liveability in the Dutch countryside (which encompasses around 30% of the Dutch population) compared different types of rural settings [11,12,13]. Statistics showed that liveability is most often challenged in small remote villages (<5000 inhabitants), especially when there is a population decline. People in these villages, compared to other villagers, are socially and economically more vulnerable and are more often confronted with problems in their living environment, such as a reduction of facilities [11,12].

Although the level of liveability does not seem to differ greatly between Dutch cities and villages [11,12,14], there is a realization that vulnerable groups, especially in small remote villages, can experience the liveability in their villages much less positively than other inhabitants [15]. Since old age increases the risk of vulnerability, these vulnerable groups for a large part consist of older people who are physically limited and/or socially isolated [11,13]. 

### 1.2. Residential Satisfaction and the Person–Environment Fit

Residential satisfaction is closely related to the concept of liveability, as it provides information about the connection between aspects of the social and spatial environment and the needs and preferences of its inhabitants [16,17]. As it is also directly related to the level of well-being of older adults [18,19,20,21], it can be considered a key outcome variable in studying ageing in place [16]. In this study, we therefore focus on mapping the level of residential satisfaction of independently living older villagers.

To gain more understanding about older people’s residential satisfaction, we use the theoretical perspective of the person–environment (P–E) fit tradition. From this perspective, residential satisfaction reflects an important part of the degree to which individual needs and preferences match with relevant aspects of the living environment. Spatial, social and functional aspects of the living environment can compensate for the limitations of (frail) older people, or even empower them, but can also raise additional obstacles [22]. The extent to which the environment “fits” a person’s skills and competencies determines (in part) the quality and the course of the ageing process and the extent to which ageing in place is and remains possible [16,23,24,25]. 

In this study, we use the P–E theory to identify the key indicators of residential satisfaction among older villagers in four small villages in the Netherlands. To this end, we take into account older people’s perceptions of spatial, social and functional attributes of the living environment. We strive to not only identify environmental indicators of residential satisfaction but also to better understand how they affect the (daily) life of older villagers. Therefore, we combine quantitative data with photovoice data that we have collected in the four villages. Photovoice, as a creative research method, is specifically suitable for mapping the more intangible aspects of people’s experiences in their living environment [26,27]. With photovoice we obtain a better picture of how people experience their living environment, in a positive and negative sense, and the emotions this evokes in them. From this perspective, photovoice can tell the story behind the numbers of our quantitative analysis.

Finally, in line with the P–E fit tradition [28], we differentiate according to the capabilities and vulnerabilities of older villagers. More specifically, we examine whether (a greater risk of) frailty is associated with a lower P–E fit and thus a lower level of residential satisfaction. If this is the case, we examine whether this effect can be (partly) attributed to a difference in people’s perceptions of the physical, social and/or functional living environment.

Our research questions read: (1) to what extent do perceptions of spatial, social and functional attributes of the living environment affect the level of residential satisfaction of older adults in four Dutch villages, and (2) to what extent is there a difference in residential satisfaction between older residents who are physically and socially vulnerable to a lesser or greater extent?

## 2. Theory and Hypotheses 

### 2.1. Liveability and Residential Satisfaction

The interaction between environmental characteristics and personal characteristics is central to the literature about “liveability” [29]. Although there is no clear definition of the concept of liveability in the literature [30,31], the concept relates to the connection between the living environment and human needs and preferences. This is clearly expressed in the definition given by Leidelmeijer et al. [32] (p. 14): “Liveability is the degree to which the living environment meets the conditions and needs that are imposed on it by people”. 

Studies often distinguish between different dimensions of liveability, indicating which environmental characteristics are relevant in predicting people’s level of satisfaction with their living environment [22,30,32,33,34,35,36,37]. Depending on the perspective from which liveability is examined, the number and nature of these dimensions differ (slightly) between studies [31]. Based on these previous studies, we distinguish in this article between older people’s perceptions of spatial, social, and functional aspects of the living environment that can affect the overall level of residential satisfaction. People’s level of residential satisfaction can, for a large part, be predicted by housing conditions, public facilities and neighbourhood characteristics [38,39]. From this perspective, residential satisfaction can be considered an important indicator of experienced liveability, as it reflects a subjective evaluation of the socio-spatial environment in which people reside [2].

### 2.2. Ageing in Place in a Rural Context

Regarding people’s (residential) well-being, the influence of the environment is increasingly being acknowledged, especially in old age. From the perspective of environmental gerontology, it is assumed that the environment has a more significant impact on people’s lives when they grow old. The socio-spatial environment is hypothesized to become more important for older adults, as their physical and cognitive capacities decrease with the process of ageing [23,40]. Socio-spatial aspects of the living environment can compensate for age-related limitations that have arisen but can also hinder older adults from undertaking (daily) activities, diminishing their autonomy and independence. 

Due to policy changes aimed at stimulating ageing in place, and a preference of older people to grow old in their own homes [41,42], stricter requirements are set for the residential environment of older adults. Older adults, with or without physical and cognitive impairments, are expected to live in their own homes for as long as possible. If the residential environment does not meet the specific needs of older adults with a higher risk of age-related vulnerability, environmental stress can occur that negatively affects their autonomy, quality of life and health status [43,44]. This assumption is reflected in the literature on ageing in place, which shows that aspects of the socio-spatial environment can negatively and positively affect the autonomy, well-being and health status of older adults [45,46,47,48].

The question arises whether this is even more the case in a rural context. Housing conditions in the Dutch countryside are supportive of ageing in place, in the sense that the often large and owner-occupied homes can be relatively easily transformed into accessible homes [49]. However, older villagers are often concerned about the maintenance of their (large) home and/or garden [50]. From the perspective of ageing in place, the limited supply of and the decline in (health care) facilities in the villages are of great concern, especially when a limited range of facilities is combined with a reduced accessibility of the surrounding villages and towns [50]. The high level of social cohesion is often mentioned as an important advantage of living in a smaller village. In accordance with this finding, villagers feel more connected with their (social) environment than city dwellers and have more local contacts [11,13,51]. However, this higher level of social cohesion in villages does not automatically result in more informal support between neighbours, when compared to urban settings [11,52]. The question therefore remains whether vulnerable elderly people in villages can benefit from these local social resources. 

### 2.3. Rural Ageing and the Person–Environment Fit

The relationship between the socio-spatial environment in which older adults live and their (residential) well-being is extensively discussed in the person–environment theories that have emerged in “ecology of ageing” and “environmental gerontology” [40,53]. Central to this theoretical framework is the hypothesis that personal capacities in combination with environmental characteristics determine the level of functioning of older adults. An optimal level can be achieved when the capacities of a person are in balance with the environmental characteristics [40]. Environmental factors can create barriers to expressing personal capacities [44] but can also compensate for a lack or deterioration of capacities [40].

Within environmental gerontology, the theoretical tradition regarding the P–E fit was applied to the target group of older adults by explicitly adding the process of ageing [40,54]. From this perspective, P–E interchange processes regarding the concepts of belongingness and agency are examined in order to understand the relationship between older adults and the social and spatial environment [40,55]. The home and the neighbourhood together are both important aspects of older people’s residential environment [56]. The more these environments meet the needs of older people, the higher the P–E fit and the higher the level of residential satisfaction. As residential dissatisfaction reflects the mismatch between personal needs and preferences and socio-spatial environmental supplies, it can be considered a relevant variable to examine the impact of personal factors, environmental factors and person–environment fit interchanges [16]. 

In community settings, environmental congruence, the fit between the person and the environment, is expressed in people’s level of residential satisfaction [16]. Although this fit may differ for (categories of) older people, (perceptions of) certain environmental attributes can be expected to negatively or positively affect residential satisfaction. On the one hand, environmental characteristics of small villages, such as a high level of social connectedness and green spaces in the living environment, can possibly increase the level of residential satisfaction of older adults and can buffer against negative effects of an age-related deterioration in capacities. On the other hand, environmental characteristics, such as a poor accessibility of the surroundings and a reduced supply of facilities, can possibly cause stress and amplify the negative consequences of age-related limitations. In this article, we therefore examine to what extent people’s perceptions of social, spatial and functional aspects of the living environment in small villages, apart from relevant personal factors, affect the level of residential satisfaction of older adults.

### 2.4. Environmental Stress among Vulnerable Older Adults 

As the P–E fit in small villages for an important part is expressed in the level of residential satisfaction, we expect the latter to differ between clusters of older inhabitants. The environmental docility hypothesis of Lawton states that “a reduction in competence, or deprived status, heightens the behavioural dependence on external conditions” [57] (p. 108). In this light, Kahana et al. [16] demonstrate that P–E fit interchanges are important in explaining individual differences in residential satisfaction. In other words, given a certain environmental context, the degree of P–E fit depends on a person’s personal capacities. From this perspective, it can be argued that physically and socially frail older adults in particular are hindered by, among other things, the limited supply of facilities and poor accessibility in small villages and for that reason experience a lower P–E fit. On the other hand, it can also be possible that especially more vulnerable people benefit more from the social resources that are present in small villages. In this study, we will explicitly test the hypothesis that age-related vulnerabilities result in a lower level of residential satisfaction. Moreover, if this is indeed the case, we test which environmental perceptions of older adults bring about this difference.

## 3. Data from the KRAKE Project

The data that we use in this study to answer our research questions have been collected as part of the Dutch–German INTERREG VA KRAKE project (203018), which focused on maintaining and strengthening the liveability, or communities’ quality of life, in small villages. In the context of the multidisciplinary KRAKE project, the researchers involved in the project, together with volunteers from local citizen initiatives, worked on optimizing the liveability and stimulating the self-management of 55 Dutch and German villages that participated in the project. Six themes related to a community’s liveability were central to the project: health care, housing, healthy lifestyle, family friendliness, local identity and services. Around each theme, a community was formed consisting of various pilot villages, local volunteers and researchers associated with different universities of applied science. The KRAKE project started in January 2016 and ended in June 2019. 

### 3.1. The KRAKE Housing Community

The housing community of the KRAKE project focused on the question of how people should organize and design the living environment of small villages/rural communities in order to optimize liveability and residential satisfaction. Researchers with a socio-spatial background carried out the research in collaboration with local initiative groups in eight Dutch and German pilot villages. The research findings, which are based on a needs assessment among villagers and a socio-spatial analysis of the living environment, were translated into (design) guidelines and strategies for local citizen initiatives and other stakeholders involved.

#### The Dutch Pilot Villages

In this article, we focus on four Dutch villages that participated in the KRAKE housing community. The villages vary from almost 1000 to approximately 2700 inhabitants. The four villages are located in the provinces of Gelderland and North Brabant.

Village one is part of a rural municipality consisting of around forty villages and rural communities. The village, which consists of two neighbouring communities that have grown together over the course of time, has around 990 inhabitants. There is a limited range of facilities, among which includes a primary school, churches, a bakery and a café. 

Village two, with around 1970 inhabitants, is the smallest village in a municipality that consists of four villages. Basic facilities, such as a supermarket, a primary school, a community centre, a church and a general practitioner (GP), are present. 

Village three has approximately 2725 inhabitants and is part of a municipality that consists of eight villages and hamlets. There are various facilities in the village, such as a supermarket, a bakery, a primary school, a community centre, a church and a GP. 

Village four is part of a relatively small municipality (15,000 inhabitants) and consists of around 1740 inhabitants. There are various facilities in the village, such as a primary school, a school for special education, a community centre, a GP and various (specialist) care institutions.

According to the typology of rural settings of the Netherlands Institute for Social Research (SCP) [11], villages one, three and four in this study can be classified as small remote villages. Village two can be labelled as a small village close to a city. 

Table 1 provides more information on the demographic and economic statistics of the four villages. As the table demonstrates, all villages have a (slight) ageing population. In addition, the percentage of younger adults in the villages decreased (slightly) in the period between 2008 and 2018. The other indicators in Table 1 give an impression of the socio-economic makeup of the four villages. Even though the average value of residential propriety (WOZ value) in the four villages is higher than the national average, we see that this value is somewhat lower in village three. In village two and three, the percentage of higher-educated people is relatively low compared to the national average, whereas this percentage is somewhat higher in villages one and four.

### 3.2. A Mixed-Methods Approach

To answer our research questions, we make use of KRAKE data that are collected by conducting mixed-methods research in the four abovementioned Dutch villages. Based on the survey data that has been collected among the households in the participating villages, we will first examine to what extent perceptions of environmental attributes are related to the overall level of residential satisfaction of older adults. Moreover, the survey data allow us to examine whether the degree of residential satisfaction differs for people who are physically and socially vulnerable and people who are physically and socially more resilient. 

In a next step, we try to interpret and understand the research findings based on the survey data, using the collected qualitative data in the villages. This qualitative data, based on photovoice studies, can give more in-depth information about older people’s perception and evaluation of the living environment. This allows us to better understand how certain aspects of the living environment affect the residential satisfaction of older villagers.

#### 3.2.1. Survey Data on Residential Satisfaction

In the four Dutch pilot villages of the KRAKE housing community, all households were approached to participate in survey research regarding residential satisfaction. The survey was distributed in the villages between January 2017 and August 2018. Within each household, one adult person was randomly asked to fill in the questionnaire: the person in the household who was older than 18 years and who was the first to celebrate his or her birthday. 

Respondents could choose whether they wanted to complete the questionnaire in writing or online. Because both the written and the online questionnaire contained an identical personal number, it was not possible for respondents to complete the questionnaire multiple times. In village two, in contrast to the other villages, respondents could only complete the questionnaire online, which is reflected in the lower response rate (see Table 2).

In the questionnaire, respondents were asked about their satisfaction with the current home, spatial aspects of public space, the social living environment (interaction with fellow villagers, connectedness) and the current range of village facilities. In addition, respondents were asked for some personal information, including the highest completed level of education, household situation and aspects of social and physical vulnerability.

Table 2 shows some background information about the survey research that was conducted in each village. As can be seen, the response rates are relatively high as compared to other survey research. This demonstrates that the level of involvement of the villagers in the KRAKE project was quite high.

Residential satisfaction refers to people’s level of satisfaction with their residential environment. Environmental conditions that affect residential satisfaction often relate to characteristics of the home or the neighbourhood. In small villages, due to the small scale, environmental conditions that affect residential satisfaction can also refer to the level of the village or township. In this study, residential satisfaction was measured with the question “to what extent are you satisfied with the living environment?”. Respondents could answer this question on a five-point scale ranging from (1). very dissatisfied to (5). very satisfied. The variable residential satisfaction is skewed to the right (skewness = −1.5), which means that a majority of the respondents are satisfied with the residential environment.

Older people’s satisfaction with their current home was measured with the variable housing satisfaction (1–5), which ranges from (1). very dissatisfied to (5). very satisfied. In addition, we included a dichotomous variable that expresses whether or not the respondent considers the current home as suitable for ageing in place (0–1). People who do not consider their current home as suitable for ageing in place received a score of one. 

In addition to perceptions of housing conditions, we included variables that relate to older people’s perceptions of spatial aspects of public space. Based on six separate variables that all relate to people’s satisfaction with the quality of public space, we constructed a new variable. Respondents were asked about their level of satisfaction with: “the natural environment”, “the maintenance of public green areas”, “paving of the public road”, “road safety”, “the maintenance of houses and buildings” and “the attractiveness of houses and buildings”. They could answer on a five-point scale, which ranged from (1). very dissatisfied to (5). very satisfied. The new variable was constructed by calculating an average score for respondents who had given a valid answer on at least four out of six variables (Cronbach’s alpha = 0.724). On the basis of three variables concerning perceived nuisance from “noise from traffic”, “dog poo” and “litter”, we constructed a new variable degree of perceived nuisance in public space. Respondents could answer on a four-point scale, which ranged from (1). no nuisance to (4). much nuisance. For the new variable, the mean score was calculated when at least two out of three variables were answered validly (Cronbach’s alpha = 0.885). The variable perceived accessibility of nearby villages and towns (0–1) indicates whether respondents perceive the accessibility of the nearby environment as mediocre or poor. The perceived accessibility of the public space was measured by presenting the following statement to the respondents: “The sidewalks in the village are a problem for people with reduced mobility or people who are in a wheelchair”. People who (strongly) agree with this statement received a score of one on this dichotomized variable. 

Older people’s perceptions of the social environment were also included. The level of connectedness to the village was measured on a five-point scale, ranging from (1). not connected at all to (5). strongly connected. Whether or not neighbours and local residents are part of a person’s potential social support network (0–1) is measured by asking respondents to whom they can appeal to, if necessary, for support. Respondents who (also) mentioned their neighbours and/or local residents in this regard received a score of one on this variable. Social nuisance is measured by asking respondents about the degree of nuisance they experience from local residents, which ranges from (1). no nuisance to (4). much nuisance.

Satisfaction with the existing facilities was measured by asking respondents how satisfied they are in general with the range of facilities in their village. The variable ranges from (1). very dissatisfied to (5). very satisfied. Moreover, respondents were asked whether they find their home and living environment suitable for ageing in place. When they answered with “no”, they were asked why. They could choose multiple answers from a list of possible options, including “no, there are not enough facilities nearby”. People who selected this answer received a score of one on the newly constructed dichotomous variable (0–1). 

Older adults’ (risk of) physical and social vulnerability was measured with four variables. The first variable indicates whether a respondent is hindered by health problems in undertaking daily activities indoors and/or outdoors (0–1). As a proxy of social vulnerability, people’s household situation is included as a dichotomous variable. This variable indicates whether the respondent is part of a single-person household (0–1). A person’s level of mobility is related to both physical and social vulnerability. Respondents who do not have a driver’s license and/or a car or who do not want to drive received a score of one on this dichotomous variable (0–1). Because age-related problems often go hand in hand with a decline in physical and cognitive capacities, we also include age in our analysis. Age was computed based on a person’s birth year and included as a dummy variable with categories (1). 55–64 years, (2). 65–74 years, (3). 75–84 years and (4). 85 years and older.

Gender, educational level and job status are included as control variables. Respondents were asked for their highest completed level of education and divided into three categories: (1). lower educated, (2). middle educated (HAVO, VWO, MBO) and (3). higher educated (HBO, WO). The variable job status is dichotomous and indicates whether the respondent is not employed in the labour market (0–1). 

For variables with a relatively high number of missing values (≥1.5%), we have included a separate dummy category for the missing values (see Table 3). For the other variables, we applied a listwise deletion. Table 3 presents the descriptive statistics of the variables that we described above.

#### 3.2.2. Photovoice 

In three of the Dutch pilot villages of the KRAKE housing community, villages two, three and four in Table 2, qualitative data were collected on residential satisfaction with the photovoice method. This participatory and visual research method is particularly suitable for collecting data about people’s experiences of “place” [58,59,60], as it can also capture more intangible aspects of place attachment, such as pride, frustration and connectedness. 

**Figure 1:**
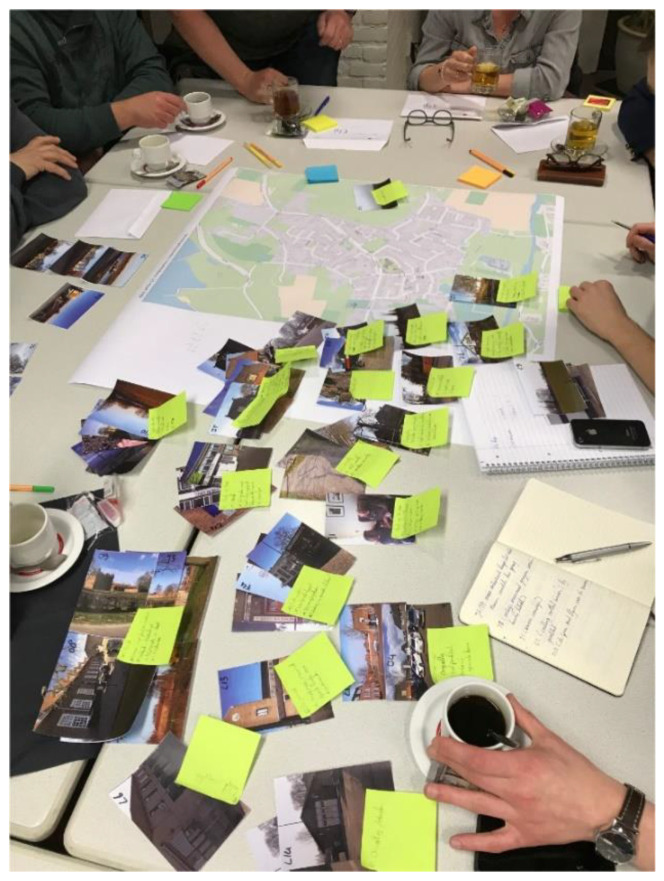
Photovoice. Source: own image.

The photovoice studies in the three KRAKE villages occurred in the time period between November 2016 and April 2018. In the three villages, a total of 60 villagers participated in these studies who differed in gender, household situation and age (although the majority was 55 years or older). The method has been applied in all three villages in a similar way, although some small adjustments were made based on previous experiences. In each village, the photovoice study consisted of three consecutive phases. During the first phase, respondents were asked to make photographs of places, buildings, objects and locations in their own residential environment that they associate with negative or positive experiences, emotions or memories. Each respondent submitted a maximum of ten photos to one of the researchers, who printed the photos and provided them with a unique code. The second phase consisted of a workshop in which the participants explained and discussed in small groups (±5–10 participants) their experiences and emotions related to place, based on the printed photos (see Figure 1). The specific buildings, objects and locations in the photos were indicated on a map of the village concerned. Each group discussion was supervised by a researcher with a background in the social or architectural sciences. The last phase of the study consisted of an expert meeting, in which the collected data, both the photos and the accompanying comments, were categorized and analyzed by 5–10 researchers with a sociological or spatial background. 

## 4. Analysis

### 4.1. Quantitative Analysis: Predictors of Residential Satisfaction

To examine the relationship between perceptions of environmental attributes and residential satisfaction, we performed a regression analysis based on 641 older adults (55+) living in the four Dutch villages (Table 4). Because of the highly skewed distribution of our dependent variable residential satisfaction, we also performed a logistic regression analysis. The results of both the linear and logistic regression analyses were strongly comparable with only a few exceptions.

In model one, in addition to the control variables, only variables related to physical and social vulnerability are included. This shows to what extent there is a difference in residential satisfaction between vital and more frail senior villagers. In subsequent models, we can see whether we can explain this effect of age-related vulnerability through perceptions of vital and more frail seniors of the physical, social and functional living environment.

Because of the ratio between the number of respondents and the number of variables, we decided to include different sets of variables successively in different models: perceptions of dwelling characteristics (model two), perceptions of the quality of public space (model three), perceptions of the social environment (model four) and perceptions of the functional environment (model five). In the final model (model six), we included all significant variables from the previous models simultaneously.

In all models, we control for gender, educational level, job status and the different variables related to physical and social vulnerability. We included a dummy variable for the village in which respondents live. 

In the first model, we tested the relationship between older adults’ (risk of) physical and social vulnerability and their level of residential satisfaction. Respondents who indicated that they are hindered by health problems in undertaking activities indoors and outdoors are less satisfied with their living environment than respondents for whom this is not the case. This implies that, as expected, the P–E fit in small villages is smaller for older adults who are physically frail. In addition to this effect, we found no other indications for the relationship between vulnerability and residential satisfaction. Respondents who cannot or do not want to drive a car are just as satisfied with their living environment as respondents who can drive a car. We also found no difference between older adults who live alone and older adults who live together (with a partner). When we controlled for physical frailty, we saw that the oldest-old do not differ from the younger respondents when it comes to their level of residential satisfaction. 

When we compared the effect of physical frailty in the different models (1–5), we saw that it sometimes decreases slightly but always remains significant. Particularly in model three, where we included variables about satisfaction with the quality of public space, we saw a decrease in the effect of physical frailty on residential satisfaction. This could indicate that people who are hindered in their daily activities due to health problems are less satisfied with their living environment overall, partly because they assess the quality of the public space as lower. However, the results of the analysis do not provide a clear explanation for the effect of physical frailty on residential satisfaction.

Model two shows that respondents who are more satisfied with their current dwelling are also more satisfied with their living environment in more general terms. The difference between respondents who are not at all satisfied with their home and respondents who are very satisfied comes down to half a point on the residential satisfaction scale (0.12 * 4). In addition to this effect, we did not find a significant difference in residential satisfaction between older adults who consider their current home as not suitable for ageing in place and older adults who have not indicated this.

Model three focuses on the relationship between people’s perceptions of public space and residential satisfaction. The variables that are included in model three relate to people’s satisfaction with the quality of public space, perceived nuisance, perceived accessibility of nearby villages and towns and perceived accessibility of the public space. The results demonstrate that older adults who are more satisfied with the quality of public space are also more satisfied with the residential environment overall. The difference between the least and most satisfied respondents regarding the quality of public space amounts to 0.8 points on the residential satisfaction scale (0.20 * 4). Moreover, the findings show that older adults who experience more nuisances in public spaces are also less satisfied with their living environment in more general terms. Both variables that relate to accessibility (of public space/of the nearby villages and towns) are not significantly related to the general assessment of older adults of their living environment. 

Variables that relate to perceptions of the social environment are included in model four. These variables in general appear to have a relatively strong relationship with older adults’ residential satisfaction. Older adults who feel strongly connected to their village scored 0.44 higher on the residential satisfaction scale (1–5) than older adults who feel strongly disconnected (0.11 * 4). Moreover, in the case that neighbours and/or local residents are part of the potential support network, older adults appear to be more satisfied with their residential environment. Social nuisance is related to residential satisfaction, in the sense that older adults who experience more nuisance from local residents are less satisfied with their living environment. 

In model five, variables are included that reflect people’s satisfaction with the functional aspects of the living environment. Older adults who are very satisfied with the existing facilities in their village scored 0.8 point higher on the residential satisfaction scale (1–5) than older adults who are very dissatisfied (0.21 * 4). Moreover, older adults who do not consider their residential environment to be age friendly due to insufficient facilities assess their living environment as less favourable.

In model six, we simultaneously included all variables that were significant in the previous models. This model shows that the effects of most variables decrease in size but remain significant. This seems logical, as people’s perceptions about different aspects of their living environment will be interrelated. What is particularly striking is that the effect of the assessment of public space is no longer significant in model six. An additional analysis shows that this is mainly due to the mutual correlation with people’s satisfaction with the available facilities in the village. The R square of model six indicates that older people’s perceptions of spatial, social and functional attributes of the living environment, together with the control variables, explain 17% of the variance in residential satisfaction. 

### 4.2. A Qualitative Interpretation of the Findings

The qualitative data that we collected with the explorative method of photovoice allow us to better understand and interpret the quantitative findings, as described above. 

**Figure 2:**
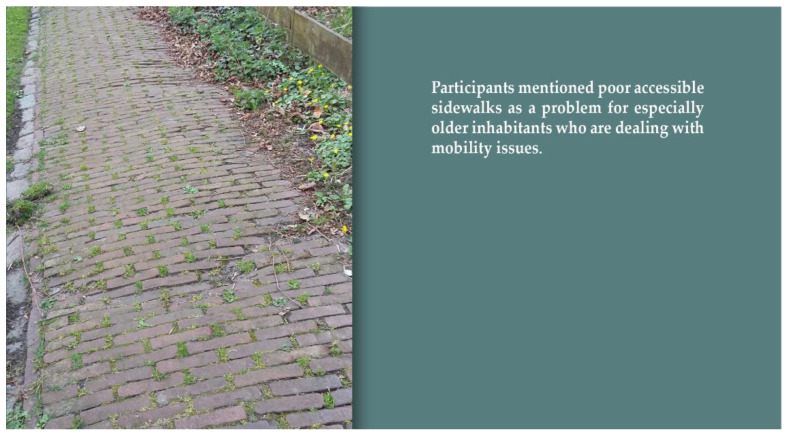
Poor accessible sidewalks. Source: photo submitted by a participant for the photovoice study.

Findings from the quantitative analysis show a somewhat confusing picture of the relationship between the perceived quality of public space and overall satisfaction with the residential environment. The relationship is initially significant but largely disappears when we control for people’s satisfaction with social and functional aspects of the living environment. However, the degree to which people experience nuisance in public space remains negatively related to overall residential satisfaction (Table 4, model six). This relationship is also clearly reflected in the results of the photovoice study. Neglected locations, such as messy entrances to the village, poor maintenance or even dilapidation of buildings and poor maintenance of the greenery were associated with feelings of dissatisfaction and frustration. Moreover, problems with the accessibility of the public space were an issue in the photovoice study, especially for respondents who are dealing with reduced mobility. In this regard, the absence or poor quality of sidewalks and cycle paths was frequently mentioned (see Figure 2). Finally, respondents were concerned about traffic safety, especially regarding speeding and dangerous crossroads. Nonetheless, the quantitative analysis demonstrates that these latter frustrations and concerns have no influence on the overall level of residential satisfaction of older residents. 

Furthermore, the findings of the photovoice study confirm the importance of perceptions of the social environment for residential satisfaction and add some in-depth information. Respondents often photographed buildings or locations that are related to the “social heart” of the village, such as the community centre or local cafe, which are often used by various (welfare) organizations and initiatives. Moreover, the (adjacent) central square in the villages was often photographed. Although the respondents were not always satisfied with the (unattractive) design of the square, it was often discussed in relation to the interconnectedness of its (various) users. Civic initiatives in the social, care and welfare domains, often represented by a specific building or location, also were often photographed and discussed by the respondents. These citizens’ initiatives often refer to services for (older) inhabitants that enable or support them to participate in community life. The joint effort by the villagers and the corresponding commitment of volunteers seem to evoke feelings of connectedness, empowerment and social engagement.

**Figure 3:**
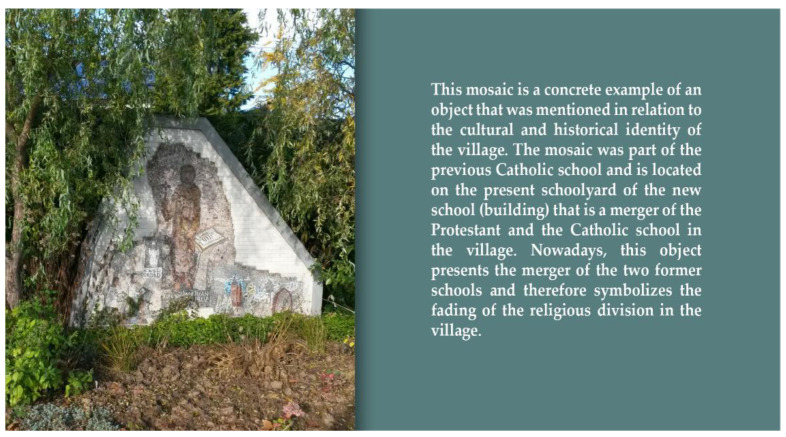
Object related to the historical identity of the village. Source: photo submitted by a participant for the photovoice study.

Moreover, the findings of the photovoice study suggest that connectedness not only has social but also spatial roots. Respondents talk about local connectedness, based on photos that refer to the identity of the village. Buildings or objects with a cultural or historical value were often photographed and discussed in relation to the identity and authenticity of the village (see Figure 3). These buildings and objects, such as a church, castle or other monument, seem to evoke feelings of pride and familiarity among the villagers. However, in some villages there is a difference between the “pearls” of the village, locations and buildings that are shared with tourists and locations, buildings or objects in the village that are especially important for the villagers themselves. In the latter case, villagers are or were often involved in the preservation and/or management. Maintaining these locations, buildings and objects is often seen as a joint concern. 

**Figure 4:**
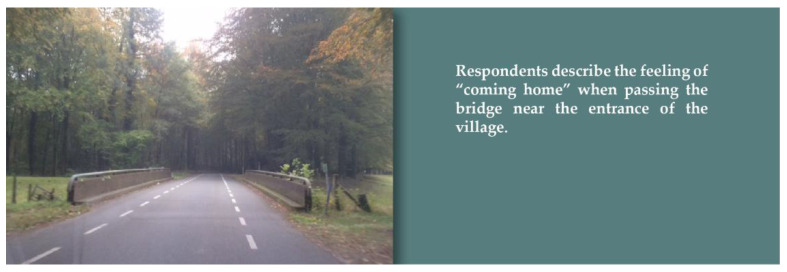
Entrance to the village. Source: photo submitted by a participant for the photovoice study.

An important spatial aspect of village identity is the entrance to the village. Respondents photographed and complained about unattractive entrances to their village, due to, among other things, litter or dilapidated buildings. However, other entrances were associated with feelings of recognition and “coming home” (see Figure 4). The entrance to the village determines, to a certain extent, the first impression people receive of the village and for that reason can make people feel welcome or, on the contrary, put them off. In addition, aspects of the natural environment surrounding the village also seem to contribute to the identity of the village. 

Our quantitative findings regarding the functional aspects of the residential environment are endorsed by findings from the photovoice study. The increasing pressure on the facilities in the villages seems to worry residents. In this context, the importance of maintaining specific facilities, such as the local supermarket, the primary school or care facilities, was often mentioned. Especially for (older) residents who are dealing with mobility issues, the accessibility of these facilities is important for maintaining independence and autonomy for as long as possible. In this perspective, respondents mentioned how disruptive it could be when older residents can no longer live in their own home and have to move to a larger village in the neighbouring area for suitable housing and care. However, facilities such as the supermarket, bakery and primary school were not only mentioned with regard to their functional character. Often, their social component was (also) discussed. After all, the informal and spontaneous contacts between residents often take place in and around these facilities and seem to be cherished by residents. 

Summarizing, we can conclude that the findings of the photovoice study illustrate the multidimensional character of connectedness, which is reflected in the social, spatial and functional layers of the living environment.

## 5. Conclusions and Discussion

In this study, we examined to what extent perceptions of environmental attributes in the living environment are related to the residential satisfaction of older adults in four Dutch villages. Based on survey data, we examined the relationship between perceptions of spatial, social and functional aspects of the living environment and the level of residential satisfaction of older villagers. Subsequently, we tried to interpret these statistical findings based on qualitative data that we collected with photovoice. This mixed-methods approach appears to be of added value, as the qualitative findings from the photovoice study help to interpret the statistical findings. Nonetheless, this approach also shows that people’s frustrations and experiences with specific aspects of their living environment, as expressed in the qualitative data, are not always significantly related to the degree of residential satisfaction in the quantitative analysis. 

First of all, the findings indicate that older adults who are more satisfied with their current home are also more satisfied with their living environment overall. However, there is no relationship between the perceived suitability of the current home for ageing in place and residential satisfaction. Regarding older adults’ satisfaction with public space, we see somewhat of a mixed picture. The degree of perceived nuisance is negatively related to residential satisfaction. People who experience more nuisance in public space are less satisfied with their living environment. Furthermore, our quantitative findings show that the relationship between the perceived quality of public space and residential satisfaction is not a direct one. This relationship is partly due to the interrelation between people’s perceptions of spatial attributes of public space and their perceptions about the social and functional attributes of the living environment. Something that is also reflected in the photovoice study, where locations, buildings and spatial objects in the village are often linked by older villagers to issues such as local identity and community formation.

Secondly, our findings show that perceptions of social aspects of the living environment affect residential satisfaction. Older adults who are more socially involved in the local community are more satisfied with their living environment, whereas older adults who experience more nuisance from local residents are less satisfied. The degree to which older adults feel connected to their village is also positively related to residential satisfaction. Additionally, the qualitative findings indicate that this connectedness to the village has social roots as well as spatial roots. Older adults value the social ties that they have in the village but also link “connectedness” to physical locations in the village that contribute to the unique character of the village and locations where people often meet. Buildings, objects and locations of historical, cultural and/or social value are often associated with the identity of the village, as well as (notable) aspects of the natural environment in which the village is located.

Thirdly, we can conclude that perceptions of the functional aspects of the living environment are also related to older adults’ residential satisfaction. Older adults who are more satisfied with the facilities present in their village (for ageing well) are also more satisfied with their residential environment overall. Qualitative data from the photovoice study show that facilities, such as care facilities and the presence of a grocery or bakery, are associated with autonomy and ageing well. Facilities are not only important for older adults because of their functional value but also because of their capacity to support spontaneous interactions with fellow villagers.

Fourthly, our research findings demonstrate that older adults who are hindered by health problems in undertaking daily activities experience a lower level of person–environment (P–E) fit in their living environment, which is reflected in a lower level of residential satisfaction. Although this relationship is strongly reflected in the analysis, our findings do not provide a clear explanation. The results show that the relationship between subjective health and residential satisfaction can only be partly explained by the way in which physically less and more vulnerable older adults perceive spatial, social and functional aspects of their living environment. Other indicators of (social) vulnerability are not related to older adults’ level of residential satisfaction. What should be noted here is that our measurement of “vulnerability” is only a proxy. After all, not all seniors who live alone at home or who are not able to drive a car can be considered “vulnerable”. Therefore, in future research, more appropriate measurements of different types of vulnerabilities should be incorporated.

In the literature that links the P–E fit framework to the ageing process [40,55], ageing well is inextricably linked to the concepts of agency and belonging. Spatial, social and functional aspects of the living environment determine, to a certain extent, the “age-friendliness” of the residential environment and for that reason are linked to ageing well. In our study of residential satisfaction, it may therefore not be a surprise that the perceptions of environmental attributes that prove to be influential for residential satisfaction relate to these concepts, in particular to belonging. Older adults’ connectedness to the village and to their fellow villagers, variables that relate to the concept of belonging, are positively related to residential satisfaction. Moreover, older adults’ satisfaction with the existing facilities in the village (for ageing well), which to some extent relates to the concept of agency, positively affects residential satisfaction. 

Finally, based on the findings of this study, we would like to make some further comments for the purpose of future research into residential satisfaction. Reasoned from the P–E fit approach, this paper provides a good picture of the extent to which older adults’ perceptions of the living environment influence their overall level of residential satisfaction. It should be noted, however, that several indicators have been measured in a generic way and are not always specified to the target group of senior villagers. This may cause our results to be a bit unspecific on certain points. For example, older adults who are less satisfied with the provision of facilities in their village are less satisfied with the residential environment overall. However, which facilities are particularly missed by senior villagers? Future research could pay more attention to detecting more targeted predictors of residential satisfaction.

In this study, we do not make a comparison between older adults who live in the city and those who live in villages. For this reason, we cannot state that predictors of older adults’ residential satisfaction will differ between urban and rural areas; in fact, they can be quite similar. However, due to demographic and societal changes, these predictors may come under pressure and the situation in smaller villages can become especially worrisome. Moreover, in small villages, older adults must continue to live at home for as long as possible, and the reduction of (care) facilities makes this increasingly difficult. First of all, this is because older adults with care needs cannot depend on (care) facilities present in their own village, but also because the reduction of local facilities can endanger the degree of social connectedness in the village. With the facilities in the villages, spots for spontaneous encounters between villagers are also disappearing. Furthermore, as we know from previous research, these spontaneous encounters, in addition to the organized encounters, are very important for promoting place attachment and public familiarity [61,62,63]. Fortunately, new housing and care concepts for older adults, designed for active ageing in rural regions, combine residential functions with social functions and health and welfare services [22]. In this regard, much attention has recently been paid to green care farms, especially in rural areas [64,65,66].

As a last comment, we would like to emphasize that the influence of the design of the spatial environment for connectedness should not be overlooked. Our qualitative data suggest that connectedness is not only the result of social aspects but also of spatial and functional aspects of the living environment. From this perspective, it seems interesting to think about the preservation of buildings and locations that are important for the identity of the village, even if they have lost their original function. Transforming, for example, churches and vacant agricultural property into housing (for older adults) seems to correspond with this idea.

## Figures and Tables

**Table 1:** Demographic and economic characteristics of the four Dutch KRAKE villages.

	Number of Inhabitants	% Population > 65 Years		% Population 25–45 Years		% Higher Educated ^1^	Average House Value (WOZ Value)	Average Standardized Household Income
	2018	2008	2018	2008	2018	2020	2018 X €1000	2018 X €1000
Village 1	990	17%	24%	22%	16%	33.3%	299	31.7
Village 2	1970	21%	24%	21%	19%	25.7%	282	30.6
Village 3	2725	14%	21%	25%	18%	20.7%	253	30.5
Village 4	1740	12%	18%	25%	21%	33.6%	283	31.5
National average		15%	19%	28%	25%	30.9%	230	29.5

Source: CBS Statline. ^1^ The statistics about educational level (population 15 to 75 years) are based on estimates.

**Table 2:** Information about the survey research that was conducted in four Dutch KRAKE villages.

	Survey	N (All)	N (55+)	Response Rate	Period
Village 1	Written and online	200	111	48%	January 2017
Village 2	Online	281	160	35%	May 2017
Village 3	Written and online	480	289	43%	April 2018
Village 4	Written and online	315	182	46%	August 2018

Source: data from the KRAKE housing community; own calculations.

**Table 3:** Descriptive statistics (unweighted) of the variables included in the analysis.

	Mean	%		Mean	%
Residential satisfaction (1–5)	4.1		Indicators of vulnerability		
			Age		
Perceptions of housing conditions			55–64 years		42.9%
Housing satisfaction (1–5)	4.2		65–74 years		40.6%
Living environment not suitable for ageing in place because of housing conditions (0–1)		16.7%	75–84 years		13.1%
			≥85 years		3.4%
Perceptions of spatial aspects of public space			Hindered by health problems in daily activities indoors and/or outdoors		
Satisfaction with quality of public space (1–5)	3.5		No		85.5%
Missing		2.2%	Yes		11.4%
Degree of nuisance in public space (1–4)	2.0		Missing		3.1%
Mediocre or poor accessibility of surroundings (0–1)		9.2%	Single-person household (0–1)		21.4%
Sidewalks problematic for people with reduced mobility			No car/driver’s license (0–1)		11.4%
Problematic		45.9%			
Not problematic/no opinion		52.5%	Background variables		
Missing		1.6%	Woman (0–1)		42.6%
			Educational level		
Perceptions of the social environment			Lower educated		38.7%
Connectedness to the village (1–5)	4.2		Middle educated		31.5%
Neighbours/local residents are part of the potential support network (0–1)		45.6%	Higher educated		27.1%
Degree of nuisance by local residents (1–4)	1.4		Missing		2.7%
			Active on the labour market (0–1)		63.8%
Perceptions of the functional environment			Yes		33.2%
Satisfaction with facilities (1–5)	3.7		No		63.8%
Living environment not suitable for ageing in place because of insufficient facilities (0–1)		15.4%	Missing		3.0%

Source: survey data from the KRAKE housing community; own calculations. N = 641.

**Table 4:** Results of a linear regression analysis of perceptions of environmental attributes on the residential satisfaction of people over 55.

	**Model 1**		**Model 2**		**Model 3**		**Model 4**		**Model 5**		**Model 6**	
	B	SE	b	SE	b	SE	b	SE	b	SE	b	SE
Intercept	4.05 ***	0.09	3.53 ***	0.18	3.66 ***	0.31	3.54 ***	0.24	3.30 ***	0.18	2.74 ***	0.36
(Risk of) vulnerability												
Age												
55–64 years (ref.)												
65–74 years	−0.09	0.10	−0.10	0.10	−0.10	0.10	−0.09	0.10	−0.07	0.10	−0.09	0.09
75–84 years	−0.20	0.13	−0.20	0.13	−0.21	0.13	−0.17	0.13	−0.19	0.13	−0.18	0.12
≥85 years	0.15	0.22	0.17	0.22	0.02	0.22	0.14	0.21	0.12	0.21	0.03	0.21
Hindered by health problems in daily activities (0–1)	−0.31 **	0.12	−0.29 *	0.12	−0.26 *	0.12	−0.33 **	0.12	−0.29 *	0.11	−0.29 **	0.11
Single-person household (0–1)	0.01	0.09	0.04	0.09	−0.04	0.09	−0.04	0.09	0.01	0.09	−0.06	0.09
No car/driver’s license (0–1)	0.06	0.12	0.06	0.12	0.12	0.12	0.05	0.12	0.08	0.12	0.08	0.11
Perceptions of housing conditions												
Housing satisfaction (1–5)			0.12 ***	0.04							0.08 *	0.03
Living environment not suitable for ageing in place because of housing conditions (0–1)			0.03	0.09								
Perceptions of spatial aspects of public space												
Satisfaction with quality of public space (1–5)					0.20 **	0.07					0.11	0.07
Degree of nuisance in public space (1–5)					−0.14 **	0.05					−0.13 *	0.05
Mediocre or poor accessibility of surroundings (0–1)					−0.18	0.13						
Sidewalks problematic for people with reduced mobility (0–1)					0.09	0.07						
Perceptions of the social environment												
Connectedness to village (1–5)							0.11 **	0.04			0.08 *	0.04
Neighbours/local residents are part of the potential support network (0–1)							0.21 **	0.07			0.16 *	0.07
Degree of nuisance by local residents (1–4)							−0.13 **	0.04			−0.10 *	0.04
Perceptions of the functional environment												
Satisfaction with facilities (1–5)									0.21 ***	0.04	0.17 ***	0.04
Living environment not suitable for ageing in place because of insufficient facilities (0–1)									−0.28 **	0.09	−0.24 **	0.09
Control variables												
Woman (0–1)	−0.02	0.07	−0.03	0.07	−0.03	0.07	0.00	0.07	−0.03	0.07	−0.02	0.07
Educational level												
lower educated	0.07	0.09	0.08	0.09	0.10	0.09	0.10	0.08	0.04	0.08	0.08	0.08
middle educated (ref.)												
higher educated	0.10	0.09	0.08	0.09	0.07	0.09	0.13	0.09	0.10	0.09	0.10	0.08
Not active on the labour market (0–1)	0.05	0.10	0.08	0.10	0.07	0.10	0.06	0.10	0.03	0.10	0.06	0.10
Village												
village 3 (ref.)												
village 1	0.31 **	0.11	0.29 **	0.11	0.20	0.11	0.25 *	0.11	0.34 **	0.10	0.20	0.11
village 2	−0.02	0.09	−0.01	0.09	−0.05	0.09	−0.06	0.09	0.02	0.09	−0.05	0.09
village 4	−0.10	0.09	−0.10	0.09	−0.13	0.09	−0.10	0.09	0.03	0.09	−0.02	0.09
R^2^	0.053		0.070		0.094		0.097		0.107		0.170	

Source: survey data from the KRAKE housing community; own calculations. N = 641; b = regression coefficient; SE = standard error; *** = *p* ≤ 0.001; ** = *p* ≤ 0.01; * = *p* ≤ 0.05.

## Data Availability

The data presented in this study are not publicly available. Statistics and tables based on this data are available on request from the corresponding author.
